# Pulsatile Lavage Systems with High Impact Pressure and High Flow Produce Cleaner Cancellous Bone Prior to Cementation in Cemented Arthroplasty

**DOI:** 10.3390/jcm11010088

**Published:** 2021-12-24

**Authors:** Kevin Knappe, Rudi G. Bitsch, Mareike Schonhoff, Tilman Walker, Tobias Renkawitz, Sebastian Jaeger

**Affiliations:** 1 Department of Orthopedic Surgery, Heidelberg University, 69118 Heidelberg, Germany; tilman.walker@med.uni-heidelberg.de (T.W.); tobias.renkawitz@med.uni-heidelberg.de (T.R.); 2 Laboratory of Biomechanics and Implant Research, Clinic for Orthopedics and Trauma Surgery, Heidelberg University, 69118 Heidelberg, Germany; mareike.schonhoff@med.uni-heidelberg.de (M.S.); sebastian.jaeger@med.uni-heidelberg.de (S.J.); 3 ATOS Clinic, 69115 Heidelberg, Germany; rudi.bitsch@atos.de

**Keywords:** total knee arthroplasty, total hip arthroplasty, pulsatile lavage systems, cemented joint arthroplasty, cancellous bone cleaning, physical parameters

## Abstract

In cemented joint arthroplasty, state-of-the-art cementing techniques include high-pressure pulsatile saline lavage prior to cementation. Even with its outstanding importance in cementation, there are surprisingly few studies regarding the physical parameters that define pulsatile lavage systems. To investigate the parameters of impact pressure, flow rate, frequency and the cleaning effect in cancellous bone, we established a standardized laboratory model. Standardized fat-filled carbon foam specimens representing human cancellous bone were cleaned with three different high-pressure pulsatile lavage systems. Via CT scans before and after cleaning, the cleaning effect was evaluated. All systems showed a cleaning depth of at least 3.0 mm and therefore can be generally recommended to clean cancellous bone in cemented joint arthroplasty. When comparing the three lavage systems, the study showed significant differences regarding cleaning depths and volume, with one system being superior to its peer systems. Regarding the physical parameters, high impact pressure in combination with high flow rate and longer distance to the flushed object seems to be the best combination to improve the cleaning of cancellous bone and therefore increase the chances of a deeper cement penetration that is required in cemented joint arthroplasty. In summary, this study provides the first standardized comparison of different lavage systems and thus gives initial guidance on how to optimally prepare cancellous bone for cemented joint arthroplasty.

## 1. Introduction

Cemented joint replacement is a widely used, safe and standardized procedure. Excellent long-term results are achieved for patients suffering from osteoarthritis [1,2,3]. Recently, a systematic review by Bunyoz et al. has analyzed the use of fixation techniques in total hip arthroplasty (THA), summarizing the trends in ten countries between 2010 and 2017 [4]. Non-cemented fixation in primary THA varied between 24% in Sweden and 71% in Denmark, indicating that a substantial percentage of THAs is implanted with the cemented technique, although various national trends were observed. Furthermore, the risk of revision in THA using cemented fixation was lower in patients older than 75 years for almost all registries surveyed. National registries also demonstrate that more than 95% of total knee arthroplasties (TKA) are performed using bone cement [5,6,7]. Thus, the overall percentage of cemented total joint arthroplasties of the hip and the knee worldwide is high, while national differences exist.

To minimize the risk of aseptic loosening, which usually manifests itself through persistent pain [8], and achieve high long-term survival rates of implants, the cementing technique has been improved and standardized in recent decades. In TKA, standard techniques include employment of high-pressure pulsatile saline lavage irrigation as well as drilling holes into the sclerotic tibia, drying the bone and applying vacuum-mixed cement to both implant and bone [9]. Third-generation cementing techniques in THA involve aggressive rasping, using high-pressure pulsatile saline lavage irrigation, using a distal cement restrictor, applying vacuum-mixed cement using a retrograde technique into the femur via a cement gun, pressurizing the cement and inserting the stem with a distal centralizer [10]. Despite these efforts to ensure strong fixation of the transplant, aseptic loosening is the main reason for revision after cemented TKA and THA [5,11,12].

Since the quality of the bone–cement interface and the penetration depth of cement are directly related to prior cleaning of cancellous bone, high-pressure pulsatile lavage is a central part of cementing techniques. Specifically, using high-pressure pulsatile lavage for bone preparation is highly recommended to achieve a sufficient cement penetration into cancellous bone during implantation [13,14,15,16,17], thereby increasing the strength of the bone–cement interface [18,19,20,21,22]. Even the presence of blood between bone and cement can reduce the integrity of the bone–cement interface [23]. A cement penetration depth of 3–5 mm is required for stable anchorage of implants [24,25]. Failure rates increase when the cement penetration depth is lower than 2 mm [26]. In addition, within the recommended cement penetration depth of 3–5 mm, harmful thermal effects of polymerizing cement are not observed, which are described for cement penetration depths of more than 5 mm [25,27].

Although guidance on the penetration depth of cement exists, the literature lacks a standardized comparison of different lavage systems and their performance. Different pressure values are reported in the literature depending on the purpose, the area of application or the type of tissue [28,29,30]. For cleaning the cancellous bone, the reported pressure showed a range between 0.48 N/mm^2^ (70 PSI) and 0.59 N/mm^2^ (85 PSI) [31]. However, data in the literature differ between the output pressure of the lavage system and the impact pressure to the surface [32]. In addition, lavage systems are available as disposable and reusable products. Single-use products usually operate with a battery pack and different cleaning tips.

Several manufacturers offer pulsed lavage systems for this purpose, but limited data are available relating to the physical parameters and how the different systems perform in comparison to each other [33]. Another important aspect with a potential impact on the outcome of the operation is a possible decrease in the performance of different systems with ongoing duration of use. Since batteries lose their power over time, differences in performance between different powering mechanisms might exist.

The aim of this prospective experimental in vitro study was to investigate the physical parameters of impact pressure, pulse frequency and flow rate over time of two battery-driven pulsatile lavage systems and one vacuum-driven pulsatile lavage system and to determine whether or not differences in the cleaning volume and cleaning depth of cancellous bone exist in a standardized setting.

## 2. Materials and Methods

There are different types of high-pressure pulsatile lavage systems that can be used to clean cancellous bone prior to cementing in joint arthroplasty. In this study, the physical parameters of impact pressure, flow rate and frequency of three different devices were tested. One of the tested lavage systems operates with a new type of vacuum drive. To test if there are differences in cleaning power, validated and standardized fat-filled carbon foam specimens, representing cancellous bone, were used.

### 2.1. High-Pressure Pulsatile Lavage Systems

**Figure 1:**
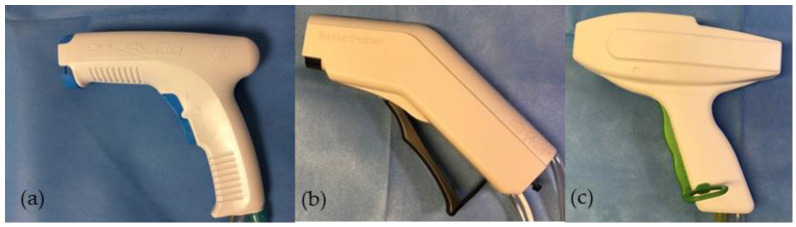
Handpieces of the three investigated pulsatile lavage systems. (**a**) Group A: Pulsavac Plus (battery powered), (**b**) Group B: InterPulse (battery powered) and (**c**) Group C: vacuum lavage.

All systems we used were single-use devices, two systems were battery powered and one new type of pulsed lavage system was driven by vacuum (Figure 1). For each lavage system, the manufacturers’ recommended bone cleaning tip was used. The lavage systems were defined as Group A—Pulsavac Plus with high capacity tibial plateau brush tip (REF 00-5150-187-00, Zimmer/Biomet, Warsaw, IN, USA), Group B—InterPulse with its bone cleaning tip (REF 0210-010-00, Stryker, Kalamazoo, MI, USA) and Group C—vacuum-lavage with the white colored knee attachment cleaning tip (development project, Heraeus Medical, Wehrheim, Germany). The lavage system of Group A uses a rocker switch to change between low- and high-pressure mode (Figure 1a). This system has been tested in both possible modes.

### 2.2. Cleaning Parameters

**Figure 2:**
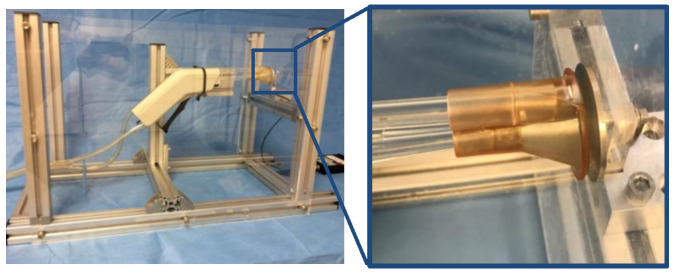
(**left**) Standardized setup with integrated lavage system to determine the impact pressure. (**right**) Close up of the round force-measuring plate on the right and the splash shield 2 mm away from it.

**Figure 3:**
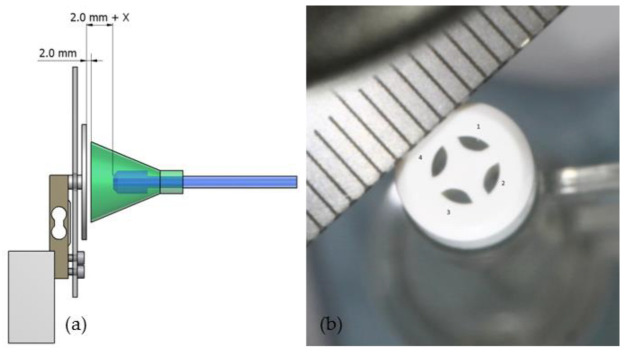
(**a**) Distance between tip and force plate, (**b**) nozzle orifice area (InterPulse shown as an example).

First, the physical parameters of impact pressure, pulse frequency and flow rate of each device were investigated. Therefore, a standardized set-up was used for each experiment as described. Each lavage system was firmly fixed on a movable, continuously adjustable slide. This allowed the splash shield to be placed at a defined distance of 2 mm in front of the force-measuring plate (Figure 2). The force was applied centrally on the force-measuring plate. The distance (2.0 mm + X) of the nozzles to the force-measuring plate was specified by the splash shield (Figure 3). This results in the distances described in Table 1 between the rinsing attachment and the surface of the force-measuring plate, including the 2 mm between the plate and the splash shield of each tip. The impact pressure was calculated by dividing the force and the area of the nozzle orifice (Figure 3). The areas of the nozzle orifice were digitized for all three investigated lavage systems using a calibrated digital microscope (Digital Microscope VHX-500 by Keyence, Osaka, Japan). Afterwards the areas were marked and calculated using the software ImageJ [34] (Table 1).

The impact pressures were determined in the course of a 30 min test. The maximum impact pressure was evaluated at 0.5, 1, 5, 10, 15, 20, 25 and 30 min after starting the rinsing process. The mean maximum was determined for an interval of 60 s at each time point of examination. For example, the mean maximum for the time from 4:30 to 5:30 (minutes: s) was calculated for the time 5 min.

**Figure 4:**
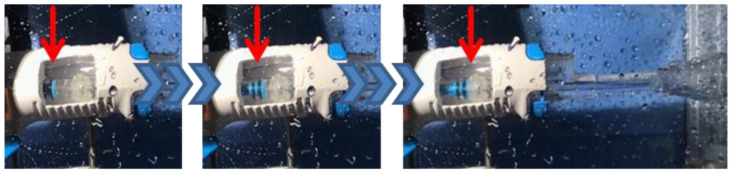
Windowed handpiece (Group A: Pulsavac Plus) to investigate the pulse frequency.

The pulse frequency of the investigated lavage system was examined using digital video analysis. The lavage system cases were windowed at the pulse-generating position in order to determine the frequency directly on the mechanical components (Figure 4). The pulse frequencies of each investigated lavage system were recorded at the same time steps as the impact pressure. The experiments were run for 30 min.

In addition, the flow rate was determined within the first 60 s after starting the process.

All tests to evaluate the physical parameters of impact pressure, pulse frequency and flow rate were run four times. Each time, a new lavage system was used.

### 2.3. Cancellous Bone/Carbon Foam Cleaning Effect

**Figure 5:**
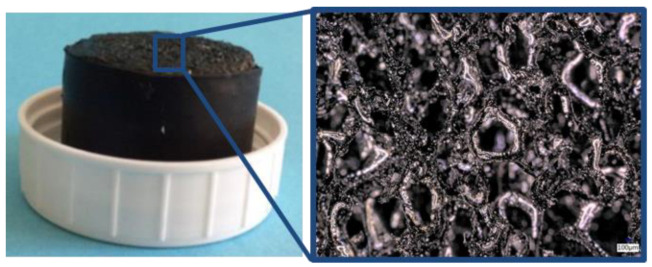
Fat-filled carbon foam specimen coated with a shrink-on tube (**left**), close up (**right**).

To determine the cleaning effect of the investigated lavage systems, we used validated and standardized carbon foam specimens (RVC foam; ERG Materials and Aerospace, Oakland, CA, USA) as substitutes for human cancellous bone [35,36,37]. Carbon specimens showed a porosity of 30 pores per inch, which correspond to 1.2 pores per millimeter. During the manufacturing process, the specimens were compressed twice, resulting in a similar trabecular bone structure (Figure 5). The carbon foam specimens were filled with standardized industrial fat (Bechem Rhus FA 37; Carl Bechem GmbH, Hagen, Germany), to simulate human bone marrow [35,36]. One ingredient of this fat is an aluminum-complex soap, which was used as the contrast medium for radiological analysis. The fat-filled carbon specimens were coated with a shrink-on tube simulating the cortex (Figure 5).

**Figure 6:**
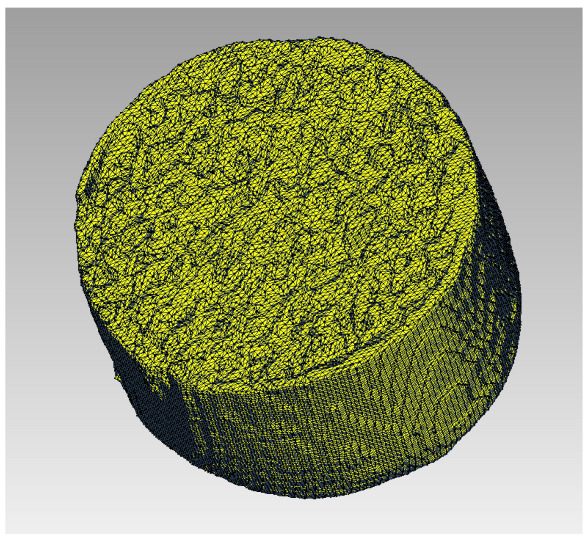
3D reconstruction of fat-filled carbon foam after cleaning.

In order to evaluate the cleaning effect, the removed fat volume and the mean cleaning depth were determined. Sample sizes of ten carbon specimens were used for each lavage system. We used ten new lavage systems in each group. For the investigation of cleaning volume and mean cleaning depth, we only used the high-pressure mode in Group A. With this standardized test setup, the lavage systems were compared using a uniform flushing volume of one liter per specimen. The cleaning distance was determined by the splash shield. The evaluation of the removed fat volume and the mean cleaning depth was analyzed using CT scans (computed tomography). The fat-filled specimens were scanned with a slice thickness of 0.75 mm before and after cleaning (SOMATOM Emotion, Siemens Healthcare GmbH, Erlangen, Germany). To determine the cleaning volume, a reference volume was recorded for each scan. The segmentation and calculation was performed with the software ITK-SNAP [38] (Figure 6). The mean flushing depth was calculated using the volume and the geometric parameters.

### 2.4. Statistic

Prior to the analysis, normal distribution of the data was evaluated using a Shapiro–Wilk test. Homogeneity of variance was verified using the Levene test. The results allowed for the use of the ANOVA test. We conducted a one-way ANOVA to assess the cleaning effects and flow rate of the lavage design. For the repeated measures ANOVA, a Greenhouse–Geisser adjustment was used to correct for violations of sphericity. The differences in impact pressure, pulse frequency, cleaning effects and flow rate between the groups were evaluated using a Bonferroni test as post hoc analysis. A repeated measure analysis of variance (RMANOVA) was conducted to test for significant differences in impact pressure depending on the measurement time point for all investigated lavage systems. Additionally, the data were evaluated descriptively using the arithmetic mean, standard deviation, minimum and maximum. The data were analyzed using SPSS 25 (IBM, Armonk, New York, USA) with a significance level of *p* < 0.05.

## 3. Results

Our study compares the physical parameters of impact pressure, flow rate and frequency of three different pulsatile lavage systems and then makes a statement about their different abilities to clean cancellous bone.

### 3.1. Impact Pressure

**Figure 7:**
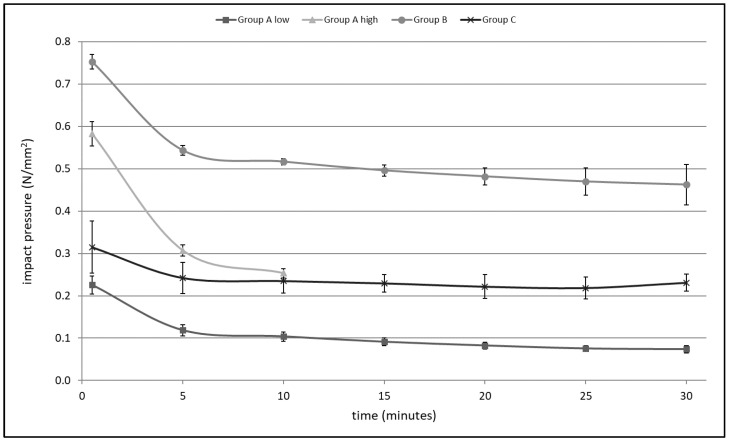
Impact pressure over time.

A significant reduction in impact pressure over time in all devices could be determined (Group A low- F(6,18) = 227.2, *p* < 0.001; Group A high- F(2,6) = 1125.3, *p* < 0.001; Group B- F(6,12) = 92.0, *p* < 0.001; Group C- F(6,18) = 12.9, *p* < 0.001) (Figure 7, Table 2). Significant differences in impact pressure were shown at all measurement times (ANOVA at 0.5 min: F(3,11) = 144.0, *p* < 0.001; at 5 min: F(3,11) = 214.6, *p* < 0.001; at 10 min: F(3,11) = 314.1, *p* < 0.001; at 15 min: F(2,8) = 561.0, *p* < 0.001; at 20 min: F(2,8) = 291.4, *p* < 0.001; at 25 min: F(2,8) = 227.4, *p* < 0.001; at 30 min: F(2,8) = 180.2, *p* < 0.001). Differences between testing times were evaluated using a Bonferroni test as post hoc analysis. This showed significant differences in impact pressure at every time point for all groups, except the 10 min time point. Here, Group A high and Group C showed no significant difference in impact pressure. In addition, a loss of impact pressure was observed when impact pressure at 30 s was defined as the reference of 100%. Impact pressure in Group A high decreased by 57% during the following 9.5 min. The other devices lost 54% (Group A low), 31% (Group B) and 25% (Group C) of their initial pressure by the 10 min point. Group B showed the highest mean impact pressure with 0.53 ± 0.02 N/mm^2^ followed by Group A high 0.38 ± 0.02 N/mm^2^, Group C 0.24 ± 0.03 N/mm^2^ and Group A low 0.11 ± 0.01 N/mm^2^ (Table 3).

For the examined system in Group A—high mode, analyses were stopped after 10 min, since all lavage systems failed due to a defect around this time point. The motor which is integrated in the handpiece continued to run, but the flushing medium was not observed. The defect occurred in four out of four tests.

### 3.2. Pulse Frequency

**Figure 8:**
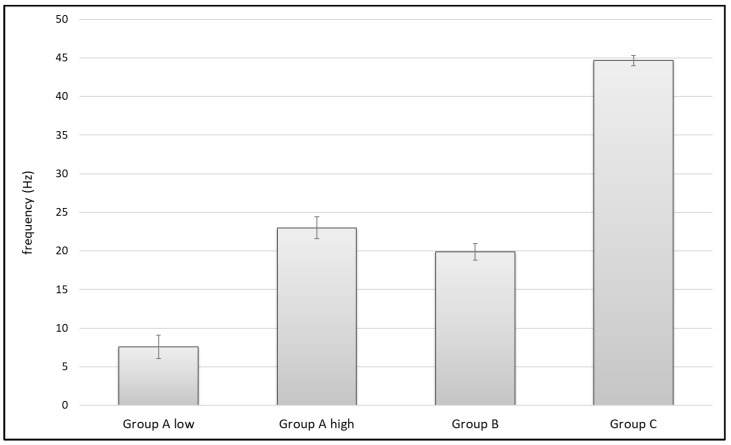
Average pulse frequency of the investigated groups.

ANOVA showed significant differences in pulse frequency between groups (F(3,21) = 1188.33, *p* < 0.001). Group A low with a mean frequency of 7.57 ± 1.51 Hz showed significantly lower pulse frequencies than the other tested devices (*p* < 0.001 in comparison to Group A high, Group B and Group C), whereas pulse frequency in Group C with a mean frequency of 44.64 ± 0.69 Hz was significantly higher compared to the other devices (*p* < 0.001 in comparison to Group A high, Group A low and Group B). Even the difference between Group A high (23.5 ± 1.53) and Group B (19.86 ± 1.07) was significant (*p* = 0.002) (Figure 8, Table 4).

### 3.3. Flow Rate

**Figure 9:**
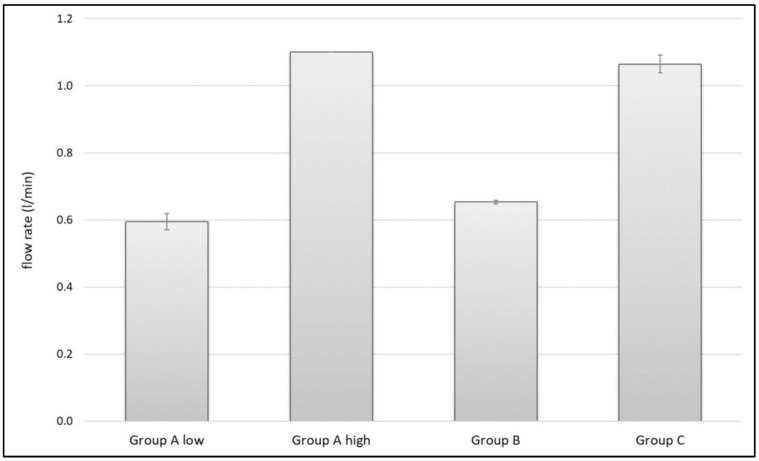
Flow rate in L/min of the different groups.

ANOVA showed significant differences in flow rate (F(3,12) = 854.9, *p* < 0.001). With a mean flow rate of 1.10 ± 0.00 L/min in Group A high and 1.07 ± 0.03 L/min in Group C, there was no significant difference between those groups (*p* = 0.114); both showed significantly higher flow rates (*p* < 0.001) compared to Group A low (0.60 ± 0.02 L/min) and Group B (0.65 ± 0.01 L/min). Even between Group A low and Group B, a significant difference of *p* = 0.007 was shown (Figure 9).

### 3.4. Cleaning Effects in Carbon Specimens

**Figure 10:**
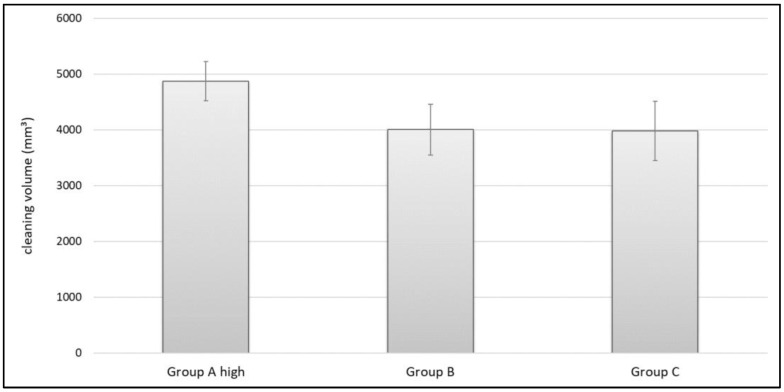
Cleaning volume of industrial fat in standardized carbon foam.

The aim of this study was to investigate differences in cleaning cancellous bone. Since the device in Group A had a rocker switch to change between low-pressure (gentle lavage for soft tissue) and high-pressure (bone preparation) we only used the high-pressure mode. When measuring the parameter of cleaning volume, Group A high showed the highest results. Its cleaning volume was 4874.2 mm^3^ (± 351.7 mm^3^). This was 870.3 mm^3^ more than Group B (4003.9 ± 452.9 mm^3^) and 896.7 mm^3^ more than Group C (3977.5 ± 532.5 mm^3^) (Figure 10). Using ANOVA, significant differences between the different groups regarding the cleaning volume were shown, F(2,26) = 12.707; *p* < 0.001. A Bonferroni post hoc analysis revealed significant difference between Group A high and Group B (*p* < 0.001). Additionally, it showed significant differences between Group A high and Group C (*p* < 0.001). No difference could be shown between Group B and Group C (*p* = 1.0).

**Figure 11:**
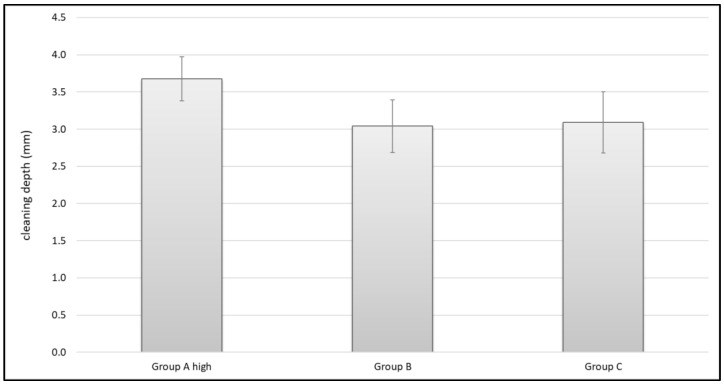
Mean cleaning depth in mm.

Looking at the cleaning depth, Group A high also showed the highest scores. Its medium cleaning depth was 3.7 mm (SD 0.3 mm). The other devices reached 3.0 mm (Group B- SD 0.4 mm) and 3.1 mm (Group C- SD 0.4 mm). All groups showed a normal distribution and significant differences regarding the cleaning depth were shown using ANOVA: F(2,26) = 10.910; *p* < 0.001. A Bonferroni post hoc analysis revealed significant differences between Group A high and Group B (*p* = 0.001) and between Group A high and Group C (*p* = 0.003). No difference could be shown between Group B and C (*p* = 1.0) (Figure 11).

## 4. Discussion

In cemented arthroplasty, high-pressure pulsatile lavage systems have an outstanding importance in preparing cancellous bone prior to cementation. In this study, we compared three different lavage systems and their cleaning effect to standardized, fat-filled carbon specimens representing cancellous bone. We were able to show that high impact pressure and high flow produced a cleaner surface, which is known to lead to improved cement penetration and therefore to a stronger bone–cement interface. This has been shown to reduce aseptic loosening [26,39], which remains the main reason for revision in TKA and THA [11,12]. A pulsed saline lavage is not the only method needed in modern cementing techniques. Drying bone by abdominal cloth and suction, drilling holes in sclerotic bone, using vacuum-mixed cement, a distal cement restrictor and filling the femur using a retrograde technique via a cement gun in THA should also be considered in cemented joint arthroplasty. Humidity, cement storage temperature, timing of cement application or cement viscosity are other aspects that have to be considered [40,41]. Even though this shows that many factors can affect cementation, cancellous bone cleaning using high-pressure pulsatile lavage has a unique importance to cement penetration and the stability of implants. Nevertheless, there are surprisingly limited data regarding the physical parameters that define pulsatile lavage systems [42]. Knowledge about comparable parameters and their impact on outcome for patients can help build empirically driven guidance on how to optimally ensure bone cleaning prior to cementation techniques in joint replacement surgery.

### 4.1. Impact Pressure

Since significant differences in impact pressure were determined between groups at different time points during the test, it appears that no common standard has been established as a reference. Only Group B reached the pressure recommended by Gross in 1971 [31]. All groups showed a significant reduction in impact pressure over time. Assuming a volume of 2 L is required to clean the bone efficiently, Group A high with the highest flow rate of 1.10 L/min processed this volume in under 2 min. Group B with the lowest flow rate (0.65 L/min) required more than 3 min to reach the recommended cleaning volume. Usually, high-pressure pulsatile saline lavage will be used for a short period of time to clean cancellous bone. In such cases, reduction in impact pressure will most likely not have clinical relevance. However, in the case of revision surgery or infections, it might be necessary to flush with higher volumes which takes more time. For example, in Group A low it takes about 10 min to flush 6 l fluid. In that case, there will be a significant reduction in impact pressure to 46% of its initial power. In contrast to the battery-driven systems, Group C is driven by a vacuum. However, a significant reduction in its performance to 75% of its initial impact pressure after 10 min was shown. Nevertheless, Group C was able to support the impact pressure the most (Figure 7, Table 5). This could be an advantage in cases where high volumes of flushing medium will be needed, such as revision surgery, septic surgery or with large traumatic wounds with serious pollution.

### 4.2. Pulse Frequency

Analyzing the pulse frequency, Group A low showed significantly lower frequency compared to the other groups. In contrast, Group C had a significantly higher frequency than all other tested devices (Figure 8). However, is there any difference in their ability to clean cancellous bone? Referring to our tests in standardized carbon foam, there is no direct correlation, since Group A high had the best results in cleaning volume and cleaning depth. If pulse frequency had an important impact on cleaning cancellous bone, we would have expected a result that was at least equivalent to the other groups. In our experiments, the system with nearly double the frequency (Group C) relative to its competitors in our test using standardized carbon foam (Group A high and Group B) did not have optimal cleaning results. This implies that a better effect when cleaning cancellous bone is not generated via higher frequencies.

### 4.3. Flow Rate

Regarding the flow rate, Group A low and Group B on one side and Group A high and Group C on the other side exhibited very different results. Both Group A high and Group C showed flow rates of more than 1 L/min, whereas Group A low and Group B showed around 0.6 L/min (Figure 9). Referring to our tests in standardized carbon foam, this might have limited impact, since Group A high and Group C showed similar flow rates but had significant differences in cleaning volume and depth. Group A high was more effective in cleaning standardized carbon foam.

### 4.4. Cleaning Effects in Carbon Specimens

Looking at the results of this study, it is clear to see that Group A high is capable of cleaning significantly more of the standardized carbon foam (representing cancellous bone) than the other two tested devices, in terms of cleaning volume and cleaning depth. Those differences cannot be explained only by one single physical parameter, because its pulse frequency, its rinsing pressure and its flow rate vary in the same range as the other two tested devices (Group B and C). Even its nozzle opening (2.1 mm^2^) that provides the flushing fluid does not differentiate it from the others. Though Group B and Group C are on different sides of the scale when considering the physical parameters mentioned above, they show a similar cleaning volume and cleaning depth. Analysis revealed that the distance between the rinsing attachment and the force plate is the single outstanding difference between Group A and its competitors. Its distance to the force plate and therefore its distance to the object that is flushed is 22.0 mm. This is 4.0 mm more than in Group C (18 mm) and 5.0 mm more than in Group B (17 mm). Cancellous bone (or the carbon foam) binds fluids and retains them. These trapped fluids might prevent the lavage from reaching deeper layers and this could result in inefficient cleaning depth. Increasing the distance between the flushing device and the surface might give fluids time to rinse out and might be useful for overcoming this “fluid saturation” effect.

One limitation to this study is the usage of an established standardized carbon foam model [35,36,37] since it does not contain human bone marrow. To overcome this, industrial grease with comparable properties was used. With the inclusion aluminum soap, measurement accuracy via CT was better than it would have been in human bone. This brings the advantage that no inaccuracy was caused in the time between the cleaning procedure and the CT scan. In contrast, during surgery cement is applied immediately after cleaning. In our laboratory experiment, we finished all specimens before scanning them. Therefore, the more stable industrial grease was identified as an appropriate alternative. It should also be noted that cleaning depth and not cement penetration depth itself was measured. However, since cleaning effect and cement penetration are directly correlated to one another [13,14,15,16], the conclusion that deeper cleaning leads to deeper cement penetration is one major assumption of this study. Furthermore, we only tested for a flat surface which represents the situation in TKA but not in THA where round surfaces are present. As such, it remains unclear if the demonstrated cleaning effects also apply to cemented hip and shoulder arthroplasty.

Concerning cement penetration, less than 1.5 mm usually leads to higher radiolucency and micromotion [20,43]. In this study, all systems were able to reach a cleaning depth of 3 mm. Since 3–5 mm is described as the level of cement penetration that leads to beneficial results [24,25], all investigated systems can generally be recommended. Interestingly, Group B alone with its low frequency and low flow rate reached the recommended pressure [31]. However, it failed to show an optimal cleaning effect. Group C with its outstanding pulse frequency and high flow rate but relatively low pressure also did not show the best cleaning effect. A significantly better cleaning effect was shown by Group A high with a combination of relatively high impact pressure and the highest flow rate. In addition, our investigation of the different system compositions revealed that Group A high showed the longest distance to the object, determined by its splash shield. This difference in the physical assembly of the device and its possible impact on cleaning performance demonstrated how important a thorough analysis of lavage systems is in order to improve existing systems and provide guidance for their use in the context of cemented joint arthroplasty.

## 5. Conclusions

A state-of-the-art cementing technique in total joint arthroplasty includes high-pressure pulsatile saline lavage for bone preparation. This is a well described procedure with good cleaning results. We see that there are many different approaches to reaching a clean bone surface in terms of the physical parameters. In this study, we compared three different devices with different tips and their geometrics regarding their impact pressure, pulse frequency and flow rate. To investigate the clinical effect of those parameters, we set up a standardized laboratory model that involved cleaning standardized fat-filled carbon foam specimens representing human cancellous bone. All systems showed a cleaning depth of at least 3.0 mm and therefore can be generally recommended to clean cancellous bone in cemented joint arthroplasty according to current standards. However, cleaning volume and cleaning depth in standardized carbon foam were significantly higher in Group A high than they were in Group B and Group C. Our analysis revealed that high impact pressure in combination with high flow rate and longer distance to the flushed object seems to be the optimal combination for cleaning cancellous bone and may increase the chances of a deeper cement penetration which is required in cemented joint arthroplasty. Further investigation with variable impact pressure, frequency, flow rate, different cleaning tips and longer distance to the surface will be useful to determine if changes lead to a more efficient bone cleaning prior to cementing.

## Figures and Tables

**Table 1:** Distances between tip and force plate surface, and the area of the nozzle orifice.

Lavage System	Distance (2.0 mm + X)	Area Nozzle Orifice
Group A	22 mm	2.1 mm^2^
Group B	17 mm	1.0 mm^2^
Group C	18 mm	4.1 mm^2^

**Table 2:** Significant differences in impact pressure over time (* defect in Group A high).

Lavage System	*p*-Value 0.5 min	*p*-Value 5 min	*p*-Value 10 min	*p*-Value 15 min	*p*-Value 20 min	*p*-Value 25 min	*p*-Value 30 min
Group A low—A high	<0.001	<0.001	<0.001	*	*	*	*
Group A low—B	<0.001	<0.001	<0.001	<0.001	<0.001	<0.001	<0.001
Group A low—C	<0.037	<0.001	<0.001	<0.001	<0.001	<0.001	<0.001
Group A high—B	0.001	<0.001	<0.001	*	*	*	*
Group A high—C	<0.001	0.009	1.0	*	*	*	*
Group B—C	<0.001	<0.001	<0.001	<0.001	<0.001	<0.001	<0.001

**Table 3:** Impact pressure in N/mm^2^.

	30 s	5 min	10 min	15 min	20 min	25 min	30 min	Mean
Group A low	0.23	0.12	0.10	0.09	0.08	0.08	0.07	0.11 ± 0.01
Group A high	0.58	0.31	0.25	defect	defect	defect	defect	0.38 ± 0.02
Group B	0.75	0.54	0.52	0.50	0.48	0.47	0.46	0.53 ± 0.02
Group C	0.31	0.24	0.23	0.23	0.22	0.22	0.23	0.24 ± 0.03

**Table 4:** Frequency over time in Hz.

	30 s	5 min	10 min	15 min	20 min	25 min	30 min	Mean
Group A low	10	9	8	7	6	7	6	7.57 ± 1.51
Group A high	25	22	defect	defect	defect	defect	defect	23.5 ± 1.53
Group B	22	20	20	20	19	19	19	19.86 ± 1.07
Group C	46	44	44.5	44.5	44	44.5	45	44.64 ± 0.69

**Table 5:** Impact pressure in % after the first 30 s.

	30 s	5 min	10 min	15 min	20 min	25 min	30 min
Group A low	100	53	46	40	37	33	33
Group A high	100	53	43	defect	defect	defect	defect
Group B	100	72	69	66	64	62	61
Group C	100	77	75	73	70	69	73

## Data Availability

Not applicable.
